# Impact of Tumor–Stroma Ratio on the Prognosis of Colorectal Cancer: A Systematic Review

**DOI:** 10.3389/fonc.2021.738080

**Published:** 2021-11-16

**Authors:** Jinlai Gao, Zhangguo Shen, Zaixing Deng, Lina Mei

**Affiliations:** ^1^ Department of Pathology, Huzhou Maternity and Child Health Care Hospital, Huzhou, China; ^2^ School of Information Engineering, Huzhou University, Huzhou, China; ^3^ Department of Internal Medicine, Huzhou Maternity and Child Health Care Hospital, Huzhou, China

**Keywords:** tumor–stroma ratio, colorectal cancer, meta-analysis, stroma content, prognosis

## Abstract

**Background:**

It is critical to develop a reliable and cost-effective prognostic tool for colorectal cancer (CRC) stratification and treatment optimization. Tumor–stroma ratio (TSR) may be a promising indicator of poor prognosis in CRC patients. As a result, we conducted a systematic review on the predictive value of TSR in CRC.

**Methods:**

This study was carried out according to Preferred Reporting Items for Systematic Reviews and Meta-Analyses (PRISMA) 2020 guideline. An electronic search was completed using commonly used databases PubMed, CENTRAL, Cochrane Central Register of Controlled Trials, and Google scholar till the last search up to May 30, 2021. STATA version 13 was used to analyze the data.

**Results:**

A total of 13 studies [(12 for disease-free survival (DFS) and nine studies for overall survival (OS)] involving 4,857 patients met the inclusion criteria for the systematic review in the present study. In individuals with stage II CRC, stage III CRC, or mixed stage CRC, we observed a significantly higher pooled hazard ratio (HR) in those with a low TSR/greater stromal content (HR, 1.54; 95% CI: 1.20 to 1.88), (HR, 1.90; 95% CI: 1.35 to 2.45), and (HR, 1.70; 95% CI: 1.45 to 1.95), respectively, for predicting DFS. We found that a low TSR ratio had a statistically significant predictive relevance for stage II (HR, 1.43; 95% CI: 1.09 to 1.77) and mixed stages of CRC (HR, 1.65; 95% CI: 1.31 to 2.0) for outcome OS.

**Conclusion:**

In patients with CRC, low TSR was found to be a prognostic factor for a worse prognosis (DFS and OS).

## Introduction

Colorectal cancer (CRC) is the third most common type of cancer worldwide and is associated with a high mortality rate (1). The TNM (T, size of the tumor and any spread into nearby tissue; N, the spread of cancer to nearby lymph nodes; and M, metastasis) provides prognostic information and aids in informed decision making in cancer patients, including patients with CRC. However, clinical outcomes in patients with colon cancer at the same TNM stage have been shown to vary dramatically. For example, approximately 5%–25% of stage II patients experienced a disease recurrence within 5 years. Additionally, patients with stage IIB colon cancer had a worse prognosis than those with stage IIIA colon cancer (2).

TNM classification is currently based on the anatomical evaluation. However, for predictive accuracy, further prognostic and/or predictive markers are required. It is critical to determine whether the early assessment of TSR and early stratification of treatment can enhance survival in selected patients. Additional biomarkers based on tumor cell features like shape, molecular pathways, genetic alterations, cell of origin and gene expression, and tumor cell immune response have been proposed. However, their disadvantage is the high cost associated with genetic and transcriptome data compared with conventional pathological examination using microscopy, which is quick, inexpensive, and reliable. Therefore, a pathological biomarker that is simple to assess is preferred. The tumor–stroma ratio (TSR) or a high percentage of stroma can be easily quantified on conventional H&E-stained paraffin sections at the invasive front of the tumor. TSR scoring is a reliable system that has the potential to be used in everyday practice. The procedure is highly replicable, with little intra-observer variation (3).

The TSR has recently been shown to be a promising outcome prediction tool in a variety of neoplasms, including breast cancer (4), colon cancer (5), hepatocellular carcinoma (6), and esophagus cell carcinoma (7). A study observed that TSR biopsy scoring in patients with esophageal adenocarcinoma was reproducible and concluded that the definitive TSR biopsy score was an independent prognostic factor for survival (8). The UNITED study (Uniform Noting for International Application of the Tumor-stroma Ratio as an Easy Diagnostic Tool) is an ongoing international multicentric prospective study to validate the TSR prognostic significance, with the goal of recruiting 1,500 patients with stage II and III colon carcinoma from 17 hospitals in 14 countries (9, 10). A recent study concluded that the use of e-learning to instruct pathologists and pathology residents seems to be an effective method. This study also showed that the consistency of scoring improved from the training to the test and demonstrated reproducibility of TSR scoring method (11).

Prognostic indicators could aid in the early identification of disease severity and stratification, as well as the planning of therapy strategies and the design of future research. The predictive value of TSR for digestive system malignancies was investigated in a meta-analysis by Zhang et al. (12). However, they were only able to include four articles on CRC in their analysis. There is a clear need for updated evidence on the association between TSR and prognosis in patients with CRC, given the publication of multiple papers following this study in recent years. The aim of present systematic review was to summarize the evidence supporting TSR as a predictor of disease-free survival (DFS) and overall survival (OS) in CRC patients.

## Methods

This study was conducted following the guidelines for Preferred Reporting Items for Systematic Reviews and Meta-Analyses (PRISMA) 2020 guideline (13).

### Eligibility Criteria

For analysis, both observational and interventional studies were considered. Studies were considered eligible if they met the following requirements: a) studies included patients with stage I or higher CRC, in association between TSR and OS or DFS; and b) studies were on human subjects with sufficient data to extract for predictive value of TSR in CRC patients and published in the English language. We excluded studies if a) TSR was provided for a number of cancers, but data for CRC could not be separated; b) studies were published as case reports, case series, reviews, or articles with no full text available, unpublished manuscripts, and conference abstracts; and c) studies lack information on TSR with CRC.

### PICO Criteria

#### Participants

We included studies on CRC with stage I or higher.

#### Prognostic Tests

Studies provided TSR value as stroma rich (Low TSR) *vs*. stroma poor (High TSR). The prognostic factor could be examined as a categorical variable. The studies that reported and classified the cutoff value of stromal ratio of 50% or higher (high stromal content) were considered in the present systematic review. Studies that set the cutoff for the ratio of stroma less than 50% were excluded from the present systematic review to obtain the homogeneous results.

#### Comparator

Low TSR *vs*. High TSR.

### Primary Outcomes

Primary outcomes assessed were DFS and OS. Survival was defined as survival till the last follow-up or censored at last follow-up.

OS was defined as the time between the date of primary surgery and the date of death from any cause or the date of last follow-up. DFS was defined as the time from date of primary surgery until date of death of any cause or the date of first loco-regional or distant recurrence.

## Material and Methods

### Study Design

Systematic review.

### Ethical Clearance

Not required.

### Search Strategy

This systematic literature search was performed following the PRISMA 2020 guidelines. An electronic search was completed using commonly used databases PubMed, CENTRAL, Cochrane Central Register of Controlled Trials, and Google scholar. The filter for the search was applied to the English language and human subjects. The last search was conducted up to May 30, 2021. The detailed search terms are given in **Supplementary Text 1**. The reference lists of the identified studies and relevant reviews on the subject were also scanned for additional possible studies.

#### Risk of Bias Assessment

The methodological quality was evaluated by QUIPS (14, 15) modified for our review. In the “study participation” domain, the moderate risk of bias resulted from the inadequate description of study participants’ selection and inappropriate exclusion. In the “study attrition” domain, moderate or high risk of bias resulted from a low proportion of baseline population analyzed or inadequate reporting with unclear risk. In the “prognostic factor measurement” domain, moderate risk of bias was most often a result of inadequate reporting and no information for blinding.

### Data Collection and Analysis

Two reviewers retrieved all eligible studies separately based on the inclusion criteria given above. The screening of the potentially eligible articles began with the title and abstract and then progressed to the full text. Any disagreements were worked out with the help of a third reviewer. The following data were extracted in the Excel file by two independent authors: details of participants, the country from the study reported, duration of the study, stage of the tumor, the cutoff value of the TSR, and outcome measurements.

### Statistical Analysis

STATA software version 13 was used to analyze the data. A random-effects model was used to calculate the pooled hazard ratio (HR) with a 95% CI. The HR of more than 1 represents poor prognosis for OS or DFS. Pooled sensitivity and pooled specificity with 95% CI were also computed using a random-effects model. The sensitivity analysis was done to check if any study significantly dominates for computing pooled effect size. Publication bias was assessed by funnel plot, Begg’s test, and Egger’s test. The *I* ^2^ statistic was used to determine heterogeneity. This test determines whether the extent of the variation is explained beyond the chance or sampling error. *I* ^2^ of less than 50% is considered unimportant, while that of more than 50% is viewed as moderate-to-considerable heterogeneity.

### Study Characteristics

**Figure 1:**
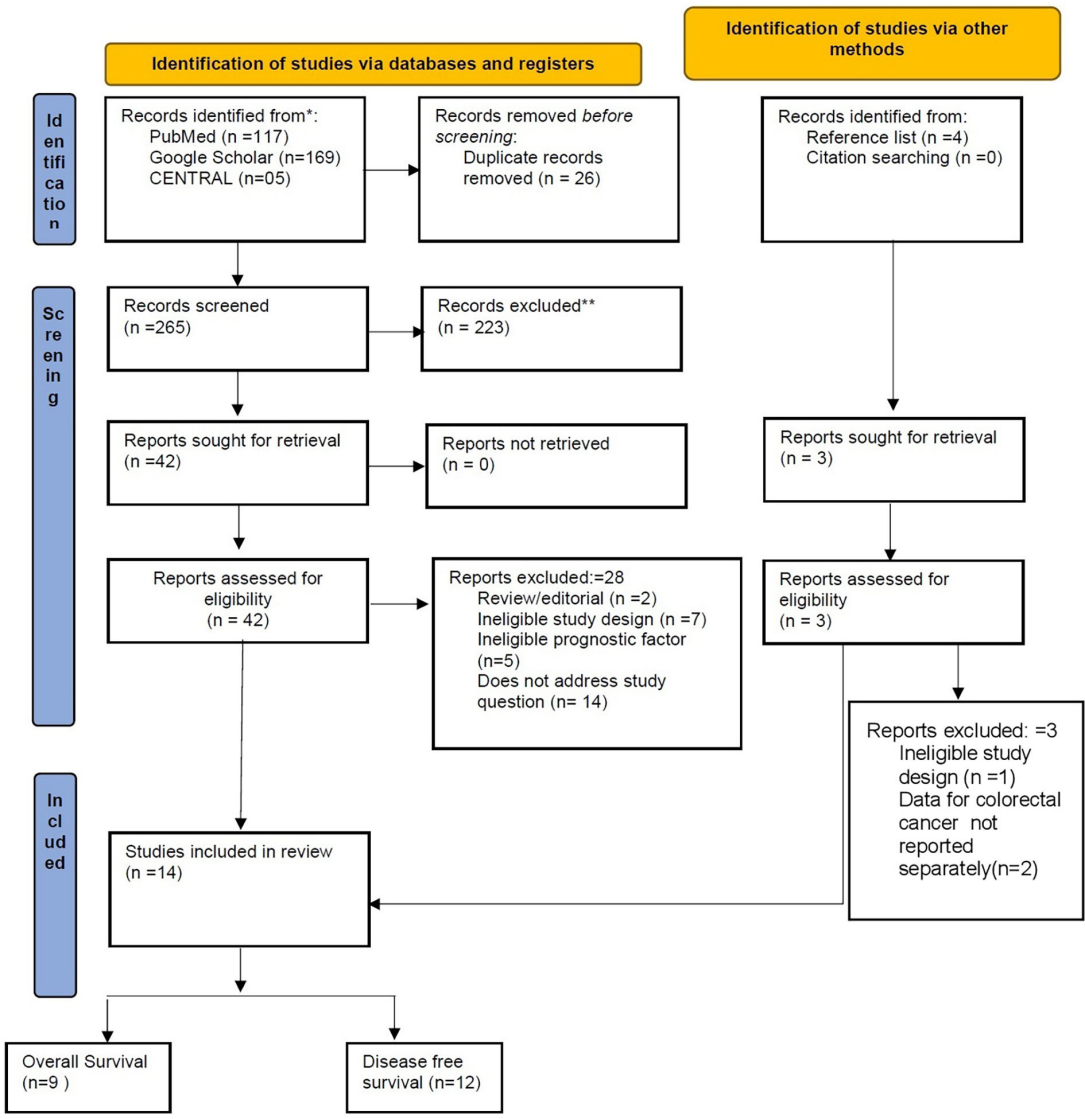
Study flow diagram representing selection process of studies.

A total of 13 studies involving 4,857 patients met the inclusion criteria for the quantitative synthesis of the present study. A study flow diagram representing the selection process of relevant studies is shown in **Figure 1**. The reason for the exclusion of the studies assessed for full text and not eligible for meta-analysis is given in **Supplementary Table 1**. The median follow-up time varied from 2.5 to 16.1 years. The mean/median age of patients ranged from 62.2 to 75 years. All the studies we included had reported the data using a cutoff value of 0.5 (50%) or more for classifying the high stroma or low TSR value. One study was reported from China (16), one from Turkey (17), three from the United Kingdom (5, 18, 19), seven from the Netherlands (20–26), one from Poland (27), and two from Denmark (28). Five studies used the retrospective study design for their study (**Table 1**). The percentage of stroma-rich cells in the included studies ranged from 12% to 65%. Two studies by the same author included the same patient group (29); therefore, the study with the higher sample size (21) was included. One study (25) did not report DFS analysis. Geessink et al. evaluated the tumor stroma ratio using both visual and automated approaches (20). Zengin et al. characterized rich-stroma as stroma more than 68% (17), while the remaining investigations used a threshold of 50%. Despite the aforementioned difficulties, sensitivity analysis revealed that no single study had a statistically significant effect on the aggregated results. For stage I CRC, only one study reported the data for association between TSR and DFS (24).

**Table 1:** Study characteristics of studies included in the systematic review.

Study no.	Author	Year	Country	Study design	Study period	Region	Cutoff	High stroma	Low stroma	CRC clinical stage	Stroma rich (%)	Median age	Follow-up	Survival	Tumor site	Treatment
1	Yang L (16)	2020	China	Retrospective	2009 to 2015	Asia	50%	35	153	II	19%	62.2	5.9	DFS + OS	Colon	Chemotherapy
2	Eriksen AC (28)	2018	Denmark	Retrospective	2002	Europe	50%	169	404	II	29.50%	73	6.9	DFS + OS	Colon	Adjuvant chemotherapy
3	Huijbers A (5)	2012	UK	Retrospective	2002 to 2004	Europe	50%	83	285	II	22.50%	75	4.3	DFS + OS	Colon	Chemotherapy and Rofecoxib
3a	Huijbers A (5)	2012	UK	Retrospective	2002 To 2004	Europe	50%	124	218	III	36.20%	75	4.3	DFS + OS	Colon	Chemotherapy and Rofecoxib
4	Mesker WE (21)	2009	The Netherlands	Retrospective	1980 o 2001	Europe	50%	34	101	I–II	27%	68.2	–	DFS + OS	Colon	Surgery
5	Roseweir AK (19)	2020	UK	Retrospective	1997 to 2007	Europe	50%	NA	NA	II	14.40%	65	5	DFS	Colorectal	Adjuvant chemotherapy
5a	Roseweir AK (19)	2020	UK	Retrospective	1997 to 2007	Europe	50%	NA	NA	III	24.90%	65	5	DFS	Colorectal	Adjuvant chemotherapy
6	Geessink OGF (20)	2019	The Netherlands	Retrospective	1996 to 2006	Europe	50%	42	87	I–III	50.30%	67	5.6	DFS	Rectal	Radiotherapy and chemoradiotherapy
7.	F. J. Vogelaar (25)	2016	The Netherlands	Prospective study	2001 to 2007	Europe	50%	124	208	I–III	37.3%	56	12	OF	Colorectal	Adjuvant chemotherapy
8	Park JH (18)	2013	UK	Retrospective	1997 to 2008	Europe	50%	67	179	I–III	24.40%	65	5	DFS	Colorectal	Adjuvant chemotherapy
9	Sandberg TP (22)	2018	The Netherlands	Retrospective cohort	1991 to 2005	Europe	50%	20	51	I–III	28.10%	67.25	5	DFS + OS	Colorectal	Surgery
10	Zunder SM (23)	2018	The Netherlands	Retrospective cohort	2004 to 2007	Europe	50%	339	824	II–III	12%		5	DFS + OS	Colon	Adjuvant chemotherapy
11	Zengin and Benek (17)	2020	Turkey	Retrospective	2004–2014	Europe	68%	78	94	III–IV	45%	76.27	8	DFS + OS	Colon	Surgery and Chemotherapy
12	Zunder SM (20)	2020	The Netherlands	Retrospective	1993–2003	Europe	50%	36	138	II	20%	64.4	15	DFS + OS	Colon	Surgery plus chemotherapy
13	Dang H (24)	2020	The Netherlands	Retrospective case cohort	2000–2014	Europe	50%	63	156	I	40%	70	3.5	DFS	Colorectal	Neo-adjuvant radiotherapy

CRC, colorectal cancer; DFS, disease-free survival; NA, Not Available; OS, overall survival.

Nine studies reported on the use of chemotherapy for treatment, with five (18, 19, 25, 28, 30) describing adjuvant chemotherapy, two (17, 26) describing surgery combined with chemotherapy, one describing chemotherapy and rofecoxib (5), and one describing only chemotherapy (16). One study (20) documented radiotherapy in conjunction with chemoradiotherapy (27), while another study (24) recommended neo-adjuvant radiotherapy in conjunction with radiotherapy as a treatment option. Only surgery was used as a treatment option in two studies (21, 22).

### Risk of Bias in Included Studies

**Figure 2:**
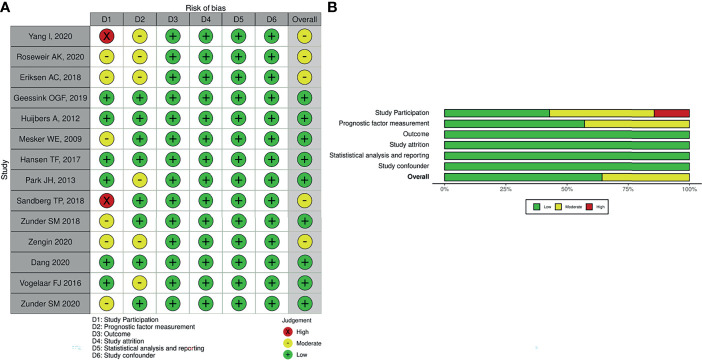
Methodological quality assessment of included studies in the present study. **(A)** Individual. **(B)** Overall.

The majority of studies were of good quality, and some concern in the study participation domains was noted in the methodological quality assessment. The methodological quality of included studies is summarized in **Figures 2A, B**. Two studies had a high risk of bias in the “study participation domain”, as they did not mention the inclusion and exclusion criteria for the study participants. These studies (16, 22) had a high risk of bias, as they did not adequately account for study participant selection, and six studies were rated as having a moderate risk of bias in study participation due to incomplete information regarding inclusion criteria of study (17, 19, 21, 23, 26, 28). Overall, we judged five studies (16, 17, 19, 22, 28) to have a moderate risk of bias. This was commonly due to study participant selection.

### Pooled Analysis

#### Disease-Free Survival

**Figure 3:**
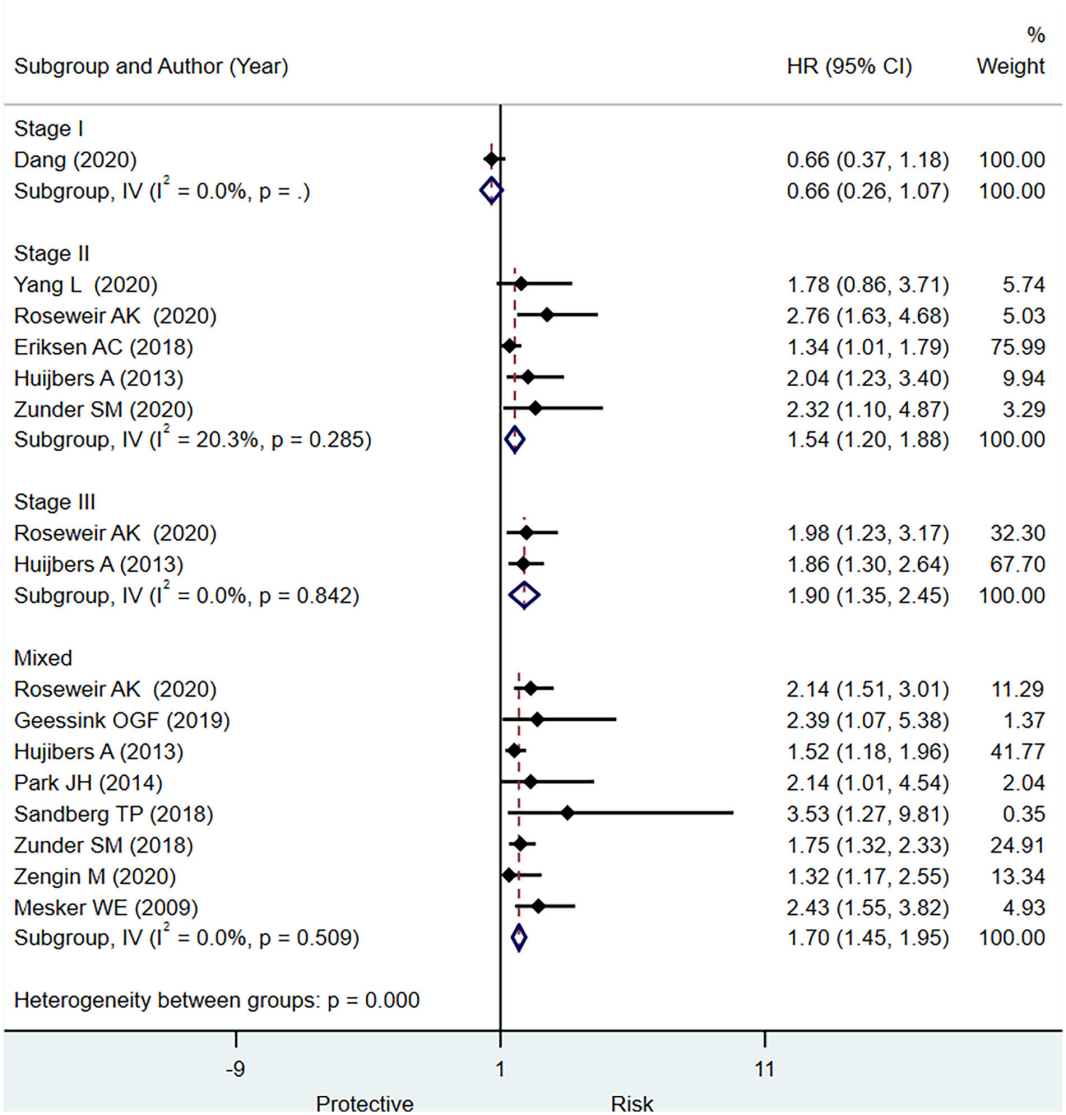
Pooled hazard ratio for different stages of colorectal cancer (CRC) for predicting disease-free survival.

Twelve studies reported the data regarding prognostic significance of TSR for DFS. In terms of the HR, we observed a statistically significantly increased risk for DFS with higher stromal content in stage II CRC, stage III, and mixed CRC patients (HR, 1.54; 95% CI: 1.20 to 1.88), (HR, 1.90; 95% CI: 1.35 to 2.45), and (HR, 1.70; 95% CI: 1.45 to 1.95), respectively (**Figure 3**). For stage I CRC, only one study did not report the statistically significant association between TSR and DFS. We did not notice a statistically significant increase in the HR for DFS in stage III CRC compared with stage II CRC (p = 0.27). These findings may be affected by type II error due to insufficient number of studies that reported data separately for stage III CRC, leading to underpowered test to examine this association. It is imperative to conduct well-designed studies to obtain precise evidence regarding the prognostic significance of TSR for predicting OS in different stages of CRC.

In terms of diagnostic test accuracy, we observed evidence of good prognostic value of low TSR for predicting worse outcome for DFS in stage II CRC with promising specificity (0.82; 95% CI: 0.76 to 0.87) but lower sensitivity (0.29; 95% CI: 0.22 to 0.37) (**Supplementary Figure 1**). The discriminative power was moderate, as assessed by pooled summary area under the curve, 0.57, 95% CI: 0.53 to 0.61 (**Supplementary Figure 2**). In case of studies including overall stages of CRC, we observed pooled sensitivity of 0.49, 95% CI: 0.30 to 0.69; a pooled specificity of 0.67, 95% CI: 0.51 to 0.79 (**Supplementary Figure 3**); and summary area under the curve of 0.62, 95% CI: 0.58 to 0.67 (**Supplementary Figure 4**)

#### Overall Survival

**Figure 4:**
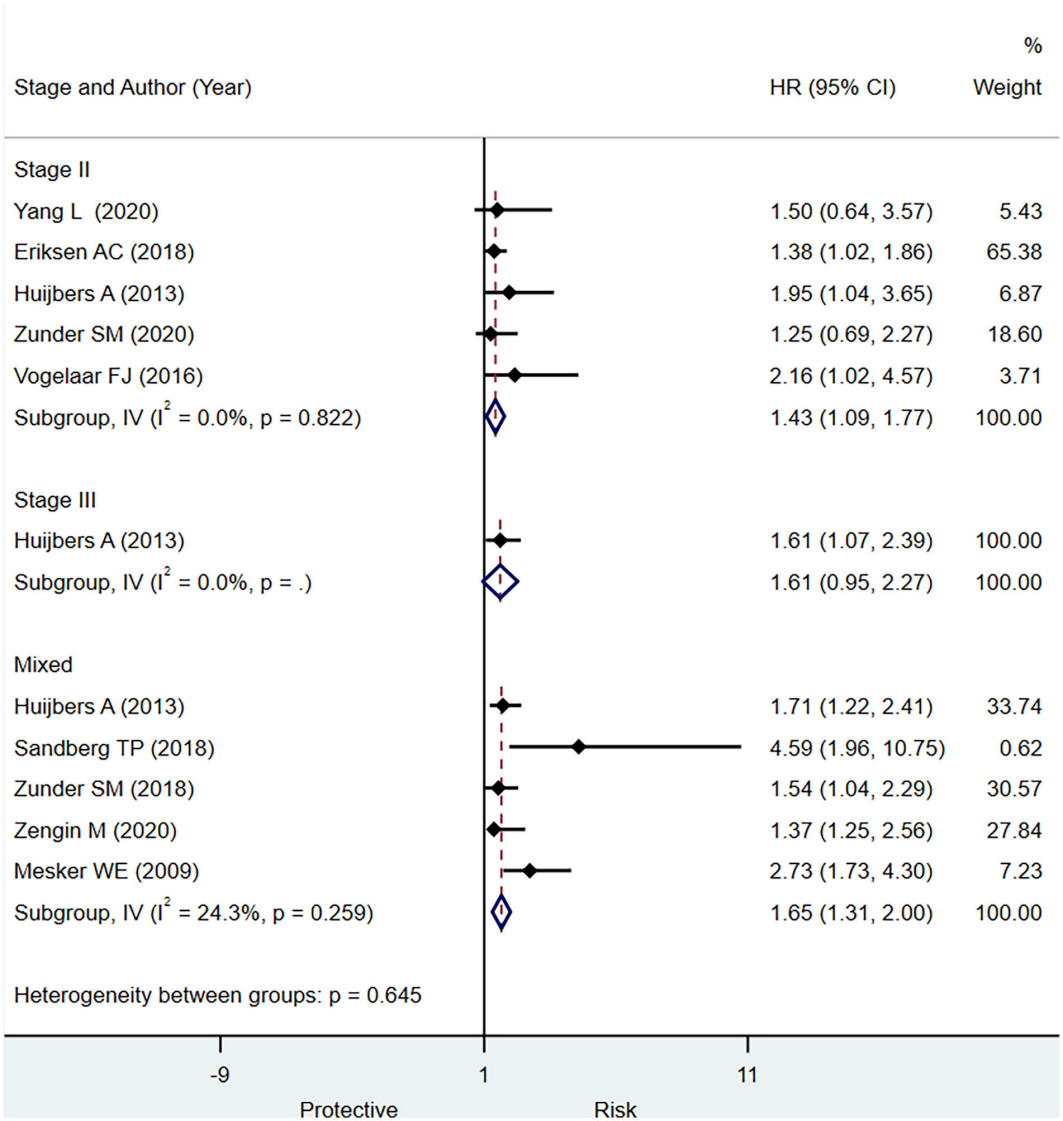
Pooled hazard ratio for different stages of colorectal cancer (CRC) for predicting overall survival.

In stage II CRC, a low TSR had a statistically significant prognostic value for OS (HR, 1.43; 95% CI: 1.09 to 1.77) based on five studies (**Figure 4**). In stage III CRC, only one study published data indicating that a higher stromal content was associated with a worse prognosis (HR, 1.61; 95% CI: 1.07 to 2.39) (**Figure 4**). Based on the pooled analysis including all stages of CRC, we observed that low TSR was significantly associated with OS (HR, 1.65; 95% CI: 1.31 to 2.00) (**Figure 4**). Five studies had sufficient data to determine the prognostic significance of TSR for OS in terms of sensitivity and specificity. We observe evidence for the good prognostic value of low TSR for predicting OS including all stages of CRC specificity (0.78; 95% CI: 0.61 to 0.89) but low sensitivity (0.49; 95% CI: 0.34 to 0.65) (**Supplementary Figure 5**) and summary area under the curve of 0.67, 95% CI: 0.63 to 0.71 (**Supplementary Figure 6**)

##### Sensitivity Analysis

**Figure 5:**
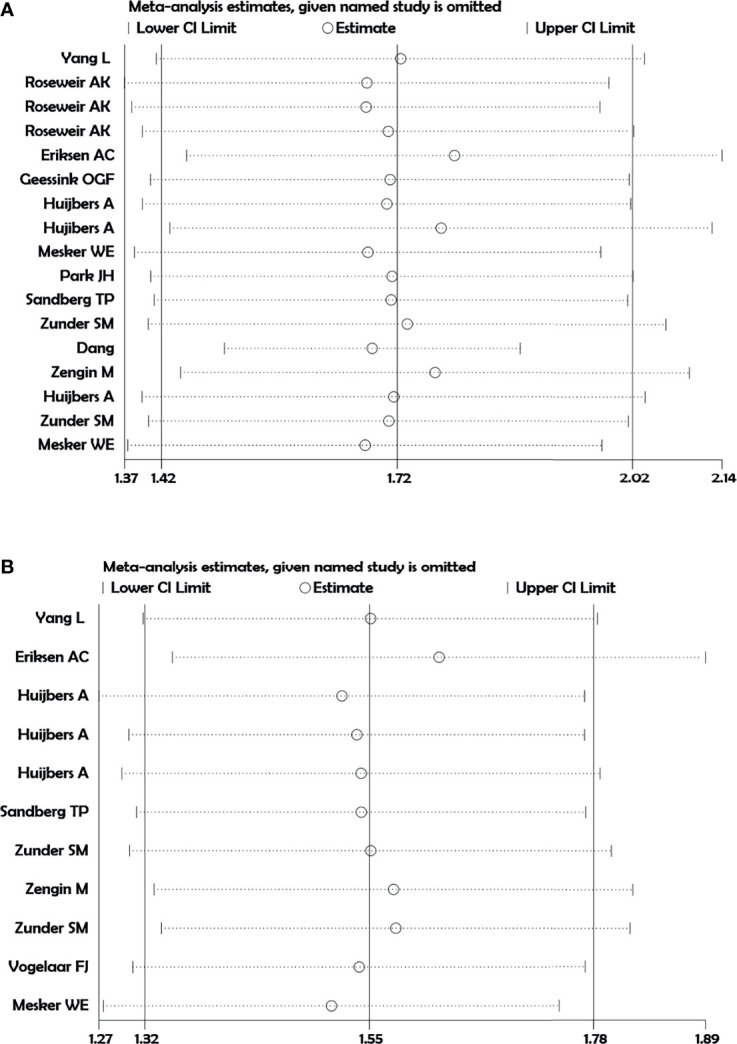
Sensitivity analysis. **(A)** Disease-free survival. **(B)** Overall survival.

We did not observe a point estimate of individual studies crossing the 95% CI of the pooled effect size of DFS and OS (**Figure 5**). This finding suggests that pooled effect size is not dominated by any individual study, indicating the homogeneity and accuracy of study results.

##### Publication Bias

**Figure 6:**
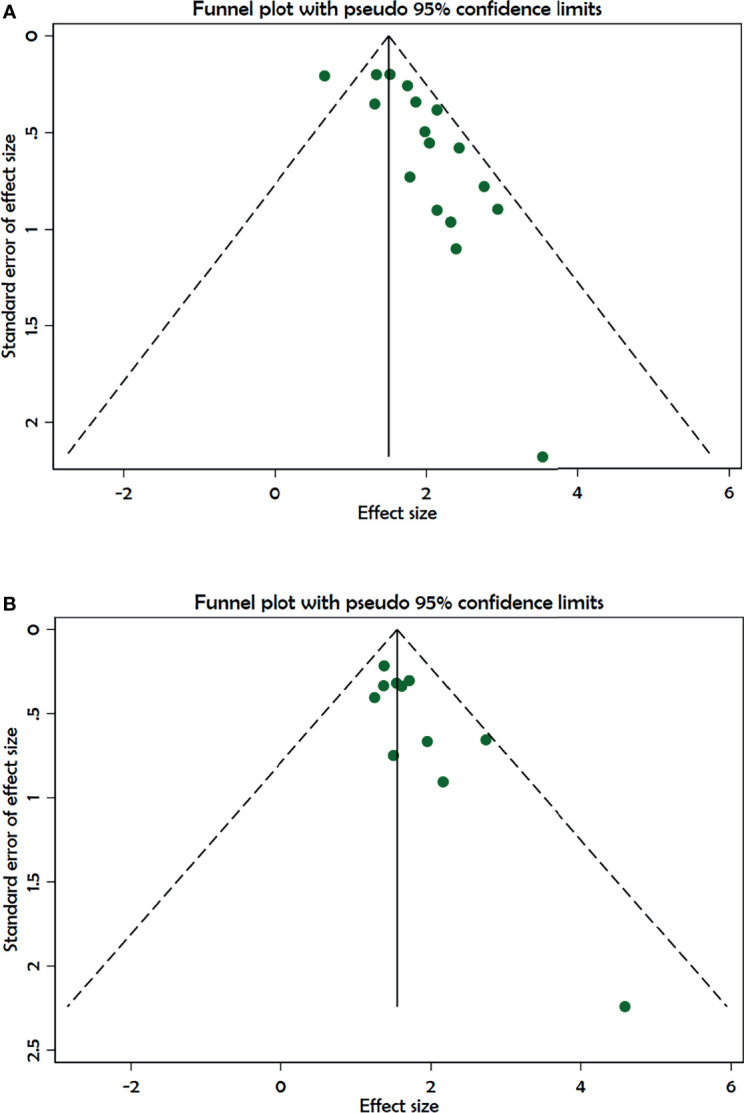
Funnel plot showing publication bias. **(A)** Disease-free survival. **(B)** Overall survival.

We observed significant publication bias for outcome DFS shown in the funnel plot and Egger’s test (p < 0.001) and Begg’s test (p = 0.32), and also for the outcome OS in Egger’s test (p < 0.001) and Begg’s test (p = 0.07); see **Figure 6**.

## Discussion

The present systematic review was conducted to summarize the evidence-based information regarding the prognostic significance of low TSR for predicting DFS and OS in patients with CRC.

There is a pressing need for an accurate, reliable, and effective approach for predicting the outcomes in patients with CRC, given the disease’s complexity in diagnosis and progression. In 2007, the carcinoma-stromal composition was discovered as the first predictive indicator for outcome in patients with CRC (29). It is simple to examine, cost-effective, and practical to use in a regular pathological laboratory. The most accepted and often utilized cutoff value for TSR is 50%, which has the highest predictive value for digestive system malignancies. A comprehensive review and meta-analysis of eight studies (four on colon or CRC) including 1,959 individuals demonstrated that a lower TSR was associated with a worse prognosis in patients with digestive system malignancies. The subgroup analysis of this meta-analysis (12) also revealed a poor prognosis for patients with low TSR in colon cancer (HR, 1.90; 95% CI: 1.51 to 2.39), which is consistent with the present meta-analysis findings; however, an association between low TSR and various stages of CRC was not presented in the same article.

In the present systematic review, we observed that a low TSR was associated with a poor prognosis in stage II CRC, and stage III CRC for OS as well as DFS. The pooled analysis revealed no evidence of substantial heterogeneity, supporting the study’s validity. Our systematic review noted that a high TSR ratio is strongly associated with a worse outcome for DFS and OS in stage III CRC as compared with stage II CRC. However, there were limited numbers of studies to draw precise conclusion for the differences in the strength of association among different stages of CRC. The study published by Yang et al. (16) consisting of low TSR proportion in 19% of study population of stage II CRC, did not observe the statistically significantly higher worse prognosis for DFS compared with high TSR subjects. On the other hand, a study published by Roseweir et al., which had a total of 14% low TSR in study population, noted statistically significantly worse outcome for DFS among low TSR subjects. The other three studies (5, 26, 28) have showed significant association of low TSR with worse prognosis for outcome DFS in stage II CRC. In the stage III CRC, two studies (5, 19) showed that low TSR is associated with worse outcome for DFS and OS.

In stage II colon cancer, four studies recruited subjects with malignancies at colon site. A study by Hujibers A et al. observed that low TSR is significantly associated with worse prognosis for DFS (HR 2.04; 95% CI: 1.23 to 3.4). The study reported by Eriksen et al. (28) and Zunder et al. (26) did not observe significant association of low TSR values with worse DFS in the stage II colon cancer subjects, whereas study by Yang et al. (16) did not observe the significant role of low TSR with worse prognosis in subjects with stage II colon cancer (HR, 1.78; 95% CI: 0.86 to 3.71). A study by Roseweir et al. (19), which recruited subjects with tumor location at colorectal site, noted that low TSR was significantly associated with worse prognosis for both stage II and stage II CRC (19). The study reported by Huijbers et al. (5) showed significant association of low TSR with both state II and stage III colon cancer patients.

We explored the effects of important clinical variables that could influence the prognostic significance of low TSR in patients with CRC, using meta-regression analysis. We observed the linear trend for different stages of CRC for higher prognostic efficiency of low TSR for predicting DFS (**Supplementary Figure 7**) but not for OS (**Supplementary Figure 8**). We cannot exclude the type II error in these findings, suggesting the power failure to detect the precise prognostic significance of low TSR value in the advance stage of CRC. Inclusion of the future studies may provide more precise information regarding the effect size of association of low TSR with different stages of CRC. Future studies should report the prognostic significance of low TSR according to the different stages of CRC to confirm these associations.

Majority of studies (9/13) used chemotherapy for the treatment. Among them, five studies (18, 19, 25, 28, 30) reported adjuvant chemotherapy. Two studies used only surgery as a treatment option (21, 22). Due to the limited number of studies in the homogeneous group, an analysis of the differences in the association of low TSR with type of therapy administered could not be conducted.

In order to investigate the relationship between clinicopathological characteristics and TSR, a meta-analysis was performed, and we found that low TSR was significantly associated with venous invasion (negative *vs*. positive OR: 0.72; 95% CI: 0.57 to 0.92, p = 0.009). However, other variables such as differentiation (moderate + well/or poor), lymph node status (positive/negative), and tumor invasion (T1 + T2/T3 + T4) were not significantly associated with TSR (31). Mesker et al. in their multivariable Cox regression analysis showed that carcinoma percentage (CP), as a derivative from the carcinoma–stroma ratio, remained an independent predictor when adjusted for either tumor stage or lymph-node status (p < 0.001 for OS). In comparison with low tumor proportion (TP) tumors, patients with high TP in colon cancer exhibited fewer tumor budding (p = 0.012), lymphovascular invasion (p = 0.049), and less harvested lymph nodes (p = 0.042) (32).

Low TSR value was substantially associated with poor survival, serious clinical stage, advanced depth of invasion, and positive lymph node metastasis, according to another meta-analysis that comprised 14 trials involving 4,238 solid tumor patients (33). A meta-analysis that included seven studies involving 1,779 patients with gastric adenocarcinoma also observed that low TSR ratio was substantially linked to 5-year rise in mortality (HR 2.19; 95% CI: 1.69 to 2.85) (34).

The underlying mechanisms of promoting the effect of low TSR on tumors are not still fully understood. According to a study, tumor-activated stroma induces epithelial cell interference, tumor invasion, and immune evasion of malignant cells (35). Tumorigenesis can be delayed or prevented by normal stroma, whereas abnormal stromal components can promote tumor growth (36). The extracellular matrix contents in the stroma promote the change in the microenvironment of the tumor that could increase the rate of tumor expansion and metastasis by overexpression of matrix metalloproteinases (37), which promotes tumorigenesis. On the other hand, the studies have shown that several growth factors and chemokines, e.g., tumor necrosis factor-alpha and nuclear factor-kB, are produced into the stroma, causing non-malignant cells to chemo-attract them. The clear-cut mechanism on the role of stromal cells for the progression of tumors has not been understood completely; however, our study showed that low TSR is associated with the progression of high-grade tumors and, thereby, poor prognosis (38).

### Limitation

Although our systematic review showed a significant role of low TSR content in poor prognosis in CRC, it does have many limitations. Few studies included in the present study had a high risk of bias in the study selection. Information for blinding of baseline characteristics to pathologists has not been provided in the many studies. Therefore, it is necessary to develop a thorough approach for evaluation. The sample size in many studies was too low for a reliable conclusion. Subgroup data on the association of gender, tumor size, tumor invasion, and lymph node metastasis was not provided in the included studies to determine the subgroup effect of prognostic significance of low TSR between these subgroups. We were unable to analyze the heterogeneity caused by differences in TNM stages, tumor sides, and cancer treatments in depth; consequently, there is an obligatory necessity to conduct studies in homogenous groups to provide credible data.

## Conclusion

The current systematic review showed that low TSR could be a prognostic factor for the prediction of worse prognosis (OS and DFS) in patients with CRC. Well-designed adequately prospective multicentric studies are required for validating the findings and for reliable conclusions on prognostic accuracy of low TSR in patients with CRC. Future research may include reporting data on the prognostic significance of TSR for each clinically relevant subgroup in order to achieve homogeneous results.

## Data Availability Statement

The original contributions presented in the study are included in the article/**Supplementary Material**. Further inquiries can be directed to the corresponding author.

## Author Contributions

JG and ZS conceived and designed the study. JG and ZD were involved in literature search and data collection. ZS and LM analyzed the data. JG and ZS wrote the paper. ZD and LM reviewed and edited the manuscript. All authors contributed to the article and approved the submitted version.

## Funding

The current study is funded by Huzhou Science and Technology Plan (Grant number: 2020GYT09).

## Conflict of Interest

The authors declare that the research was conducted in the absence of any commercial or financial relationships that could be construed as a potential conflict of interest.

## Publisher’s Note

All claims expressed in this article are solely those of the authors and do not necessarily represent those of their affiliated organizations, or those of the publisher, the editors and the reviewers. Any product that may be evaluated in this article, or claim that may be made by its manufacturer, is not guaranteed or endorsed by the publisher.
