# Supplementary material for: Impact of Tumor–Stroma Ratio on the Prognosis of Colorectal Cancer: A Systematic Review
Source: Front Oncol. 2021 Nov 16;11:738080. doi: 10.3389/fonc.2021.738080 (PMC8635241; doi:10.3389/fonc.2021.738080)
Supplement: Supplementary file 1 [file DataSheet_1.docx]

| **Study no.** | **Author** | **Year** | **Reason for exclusion** |
| --- | --- | --- | --- |
| 1 | Danielsen H. E | 2018 | No clear information was available regarding the numbers of high stroma or low stroma (TSR low & high) population. |
| 3 | Wyk HCV | 2019 | No information regarding death - survival or Disease free survival - With disease survival among TSR Low & high was available. It only reported Cancer free survival |
| 4 | Zengin M | 2019 | Heterogeneity of the cut-off values from other included study together with it includes stage 1, No clear information regarding OS,DFS , Death & Survival, cut off of 68% was used. |
| 5 | Scheer R | 2017 | Data according to desired cut-off value is not reported |
| 6 | Falm et al | 2017 | Data on hazard ratio was not reported, No separate survival data till 7 year of follow up was repoted. |
| 7 | Zhao K | 2020 | Model based on artificial intelligence quantified tumor –stroma ratio |
| 8 | West | 2010 | Relevant data on OS and DFS not reported, Cut off of 47 were reported |
| 9 | Martin B | 2020 | Insufficient data |
| 10 | Hansen TF | 2017 | Hazard ratio was not available to compute |

Supplementary table 1. Excluded studies after the full text review

**Search terms**

The following free text words and medical subject heading (MeSH) terms were used : ("Stromal ratio"[All Fields] OR ("stroma"[All Fields] OR "stromas"[All Fields]) OR (("cysts"[MeSH Terms] OR "cysts"[All Fields] OR "cyst"[All Fields] OR "neurofibroma"[MeSH Terms] OR "neurofibroma"[All Fields] OR "neurofibromas"[All Fields] OR "tumor s"[All Fields] OR "tumoral"[All Fields] OR "tumorous"[All Fields] OR "tumour"[All Fields] OR "neoplasms"[MeSH Terms] OR "neoplasms"[All Fields] OR "tumor"[All Fields] OR "tumour s"[All Fields] OR "tumoural"[All Fields] OR "tumourous"[All Fields] OR "tumours"[All Fields] OR "tumors"[All Fields]) AND "Stromal"[All Fields] AND ("ratio"[All Fields] OR "ratio s"[All Fields] OR "ratioes"[All Fields] OR "ratios"[All Fields]))) AND ("colorectal neoplasms"[MeSH Terms] OR ("colorectal"[All Fields] AND "neoplasms"[All Fields]) OR "colorectal neoplasms"[All Fields] OR ("colorectal"[All Fields] AND "cancer"[All Fields]) OR "colorectal cancer"[All Fields]).


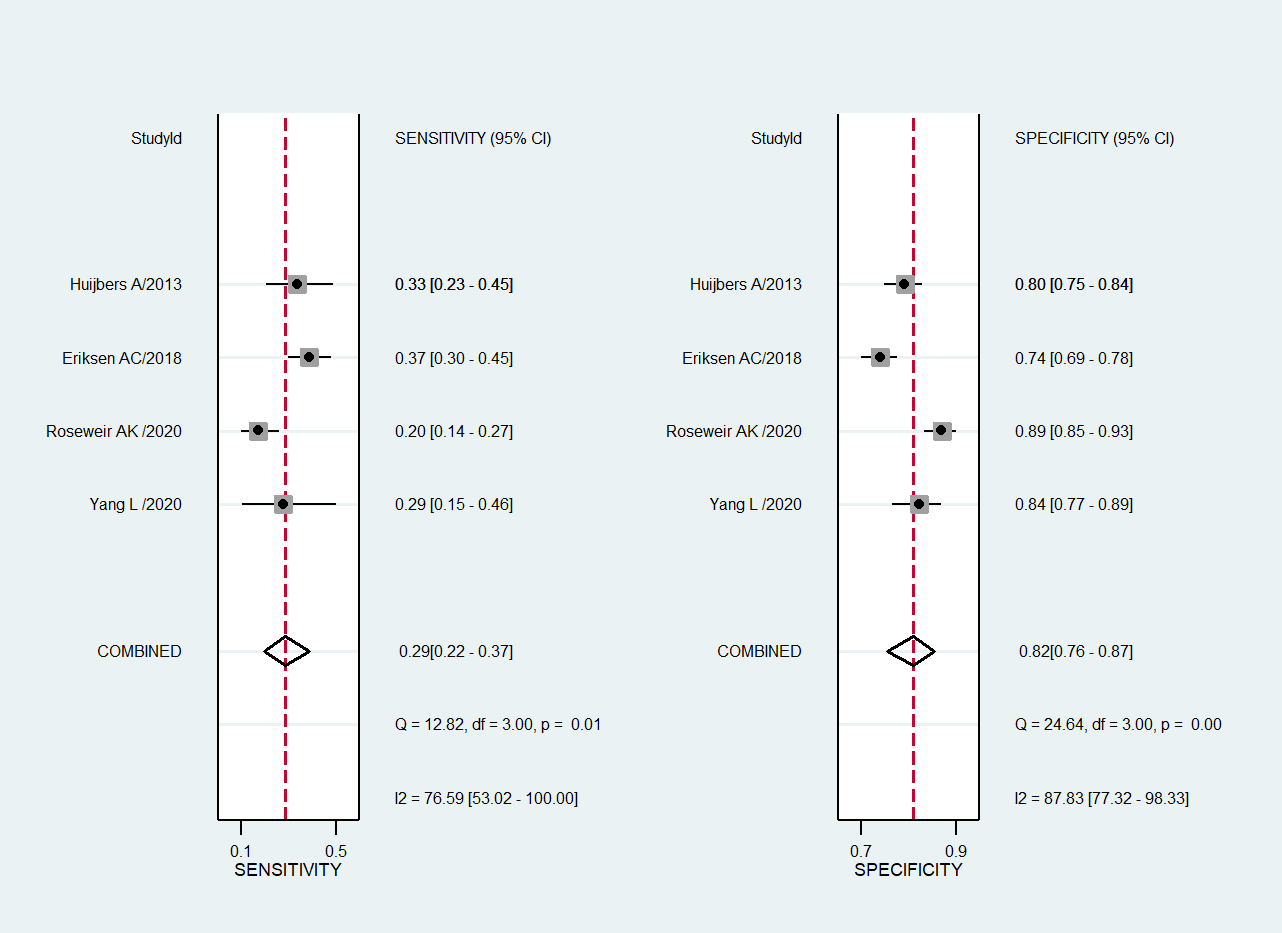


Supplementary Figure 1: Pooled sensitivity of TSR for predicting disease free survival in stage II CRC


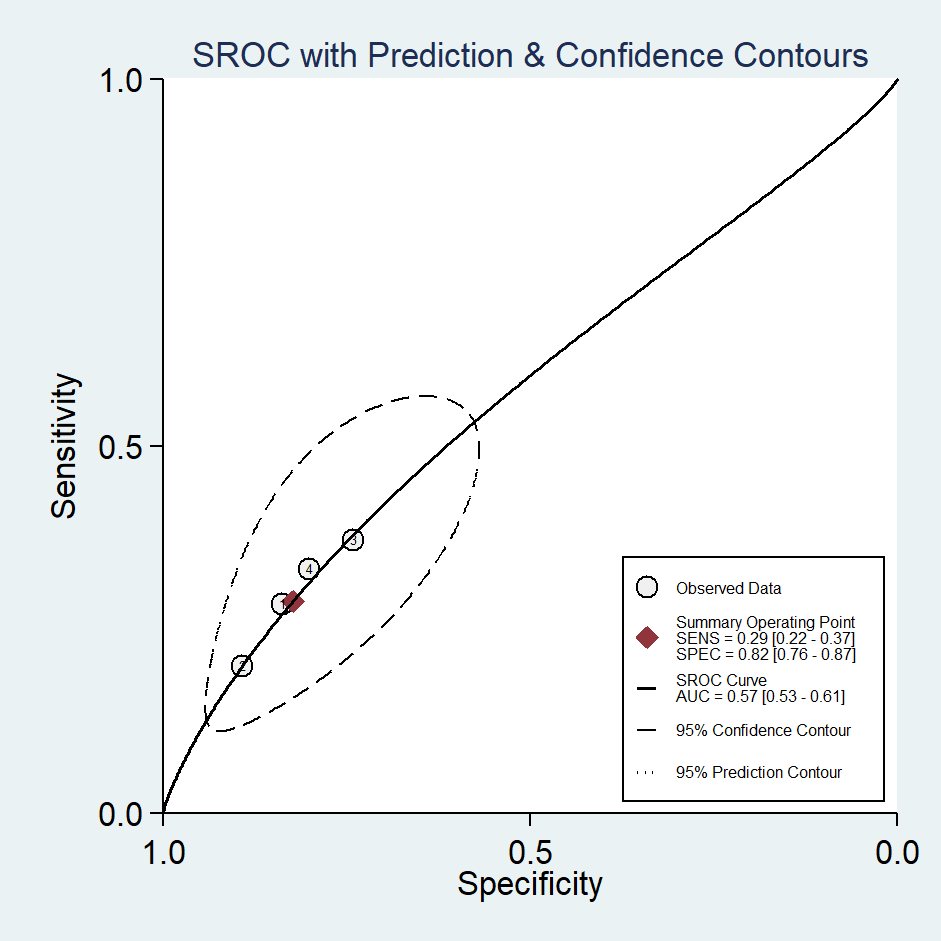


Supplementary Figure 2: SROC for discriminating DFS using TSR in stage II CRC


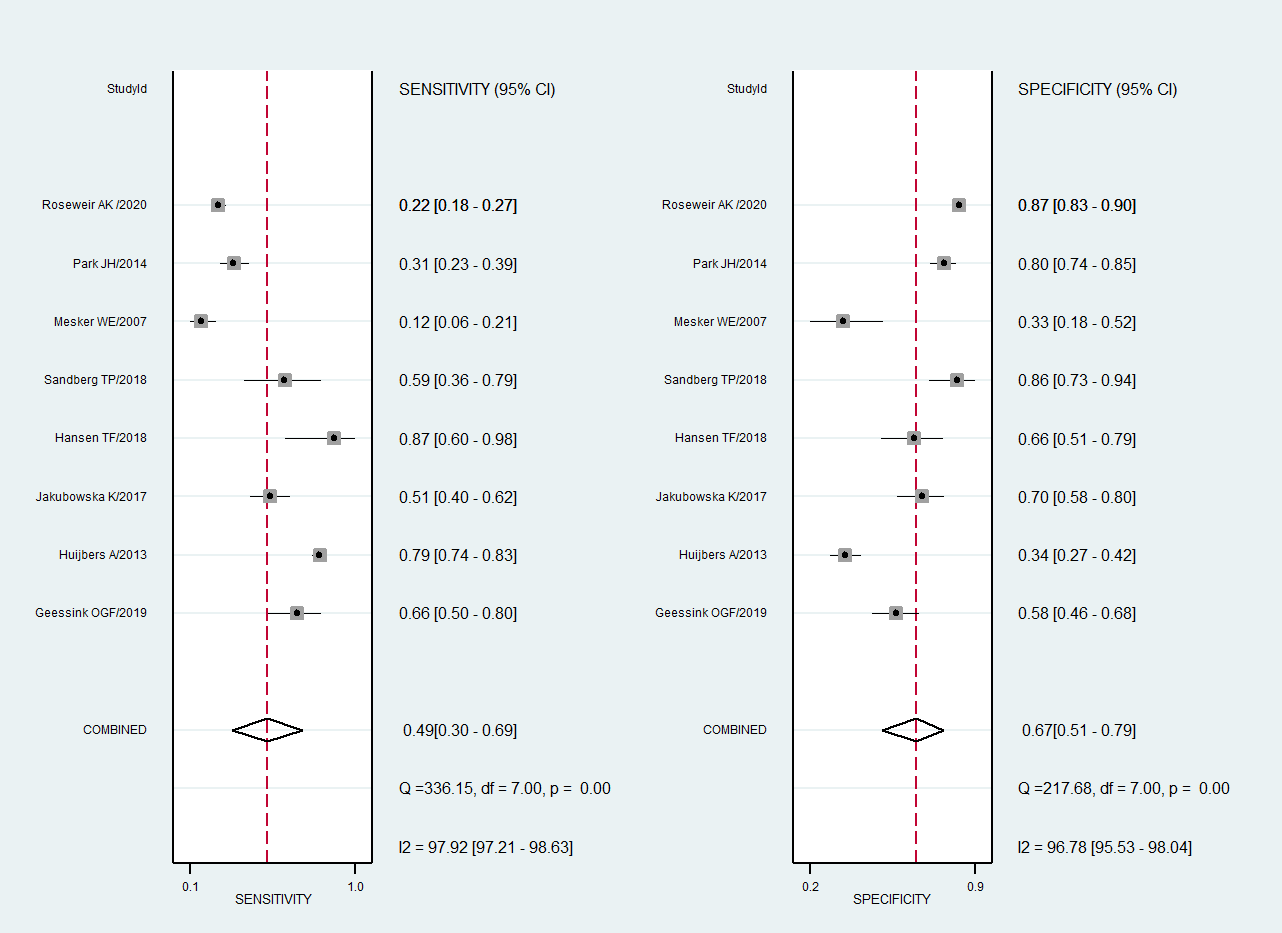


Supplementary figure 3: Pooled sensitivity of TSR for predicting disease free survival in mixed stages CRC


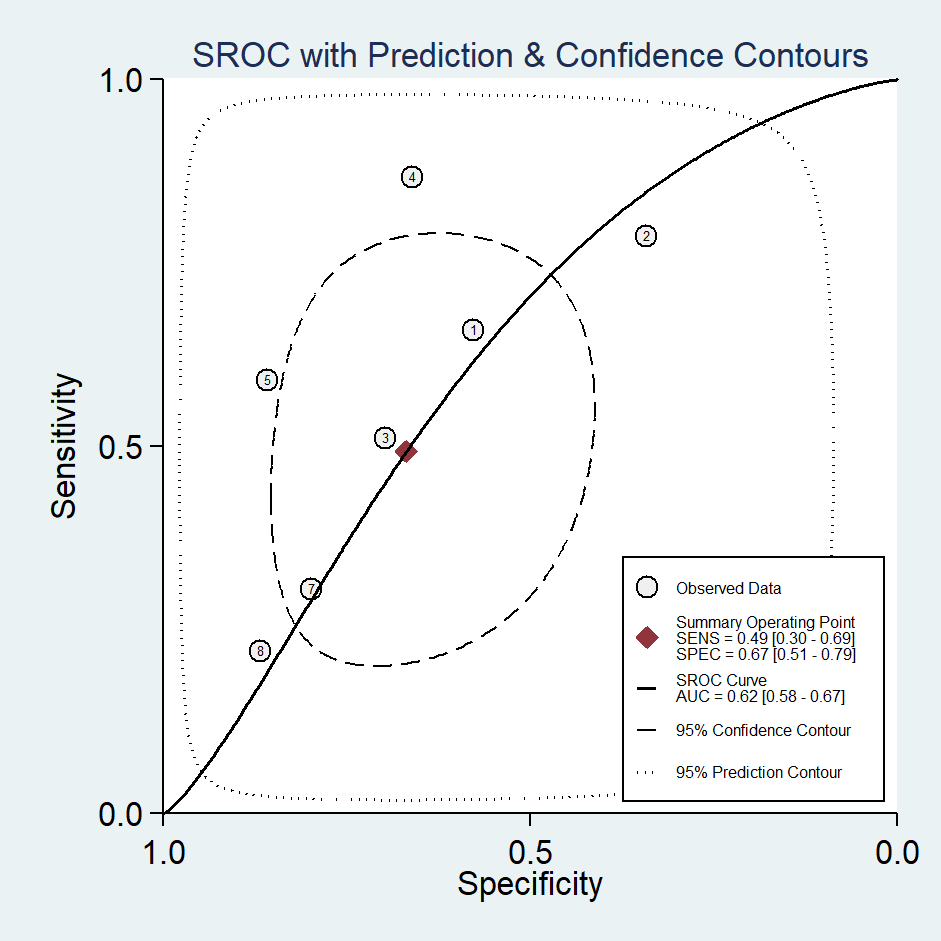


Supplementary Figure 4: SROC for discriminating DFS using TSR in mixed stages of CRC


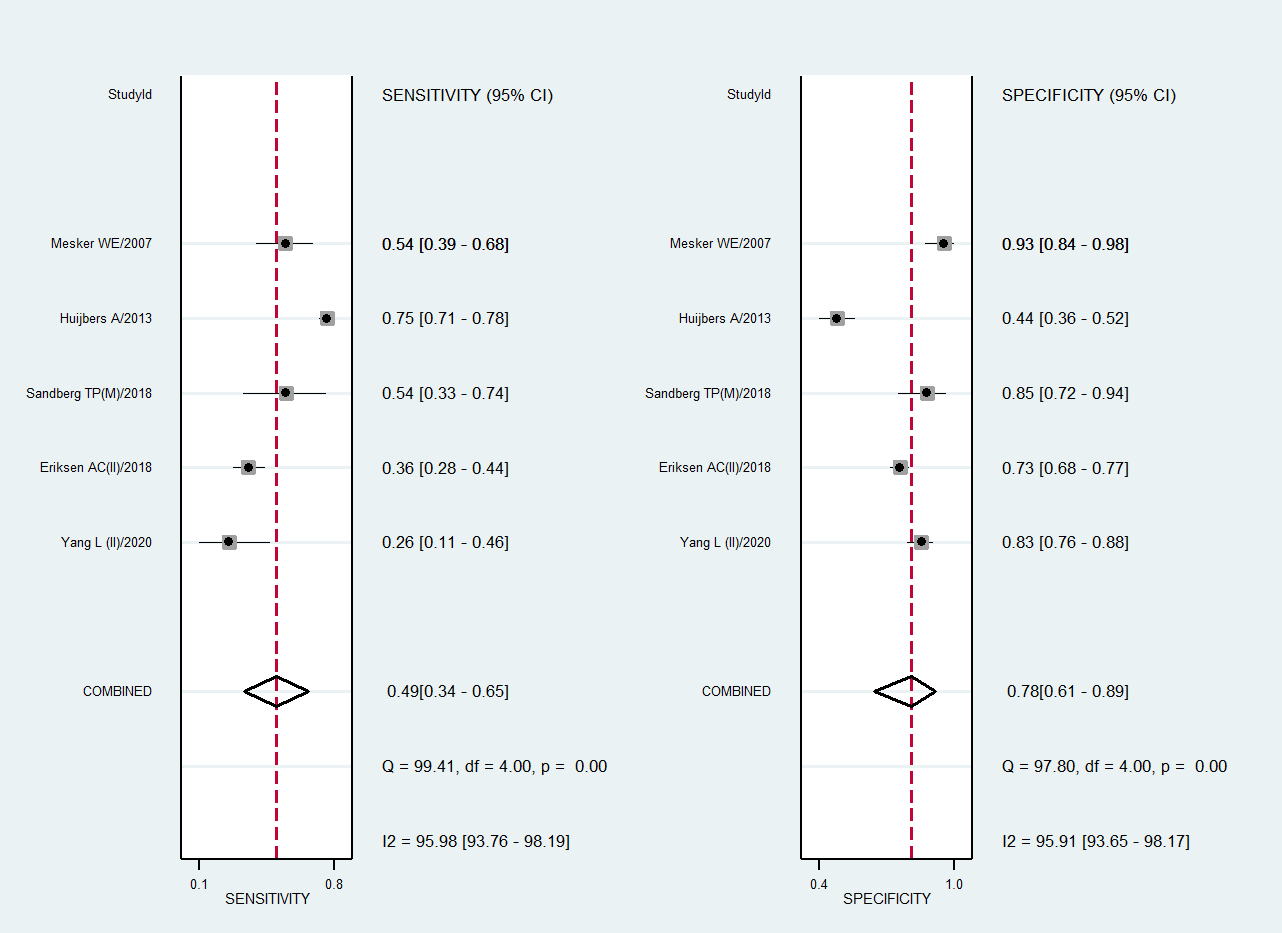


Supplementary figure 5 : pooled sensitivity and pooled specificity for predicting overall survival all stages


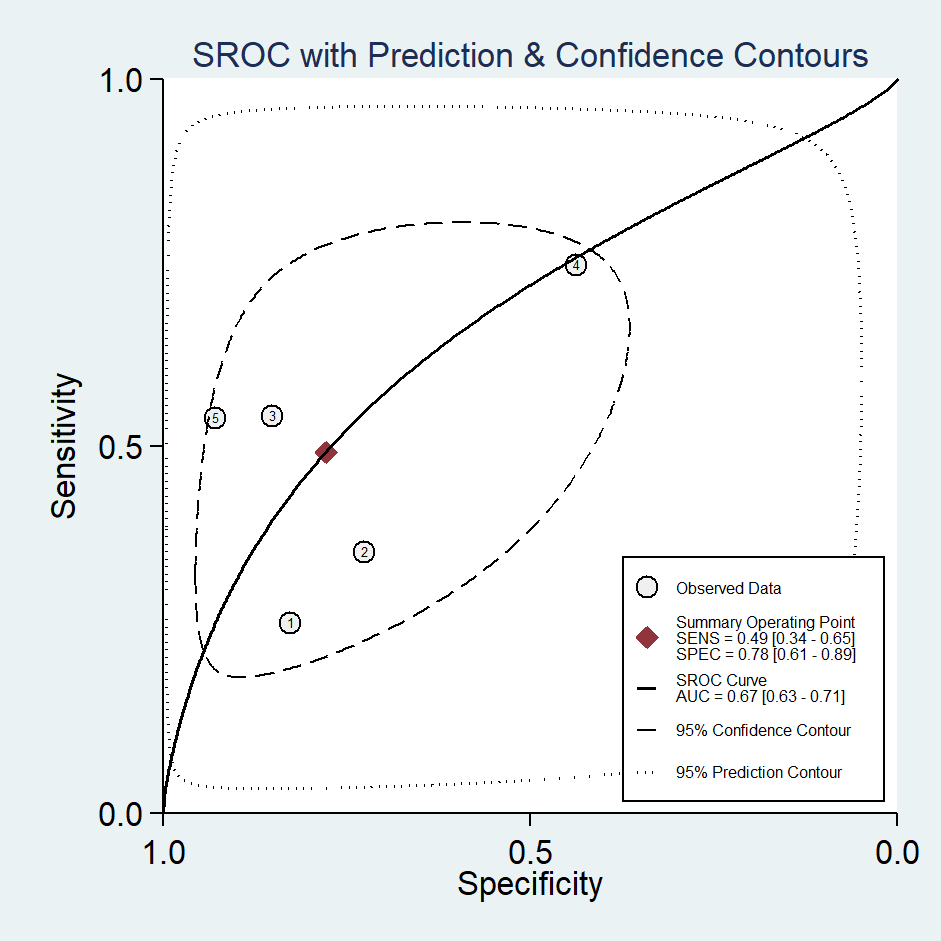


Supplementary Figure 6: SROC for discriminating OF using TSR in mixed stages of CRC

SROC all stages

Supplementary Figure 7: Meta-regression analysis showing variation of prognostic significance among different subtypes of cancer our outcome disease free survival

P value = 0.009

Supplementary Figure 8: Meta-regression analysis showing variation of prognostic significance among different subtypes of cancer our outcome disease free survival

P =0.65
